# Italian Expert Consensus on Clinical and Therapeutic Management of Multiple Chemical Sensitivity (MCS)

**DOI:** 10.3390/ijerph182111294

**Published:** 2021-10-27

**Authors:** Giovanni Damiani, Marco Alessandrini, Daniela Caccamo, Andrea Cormano, Gianpaolo Guzzi, Andrea Mazzatenta, Alessandro Micarelli, Alberto Migliore, Alba Piroli, Margherita Bianca, Ottaviano Tapparo, Paolo Daniele Maria Pigatto

**Affiliations:** 1 Department of Biomedical, Surgical and Dental Sciences, University of Milan, 20122 Milan, Italy; dr.giovanni.damiani@gmail.com; 2 Clinical Dermatology, IRCCS Istituto Ortopedico Galeazzi, 20161 Milan, Italy; 3 PhD Degree Program in Pharmacological Sciences, Department of Pharmaceutical and Pharmacological Sciences, University of Padua, 35131 Padua, Italy; 4 Department of Clinical Sciences and Translational Medicine, University of Rome Tor Vergata, 00133 Rome, Italy; malessandrini63@gmail.com; 5 Department of Biomedical Sciences, Dental Sciences, & Morpho-Functional Imaging, Polyclinic Hospital University, 98124 Messina, Italy; daniela.caccamo@unime.it; 6 International Society of Doctors for Environment, 82100 Benevento, Italy; cormano_andrea@libero.it; 7 Italian Association for Metals and Biocompatibility Research—A.I.R.M.E.B., 20122 Milan, Italy; gianpaolo_guzzi@fastwebnet.it; 8 Department of Neuroscience, Imaging and Clinical Science, ‘G. d’Annunzio’ University of Chieti-Pescara, 66100 Chieti, Italy; amazzatenta@yahoo.com; 9 Institute of Mountain Emergency Medicine, Eurac Research, 39100 Bolzano, Italy; micarel@dia.uniroma3.it; 10 ITER Center for Balance and Rehabilitation Research (ICBRR), 02032 Rome, Italy; 11 Department of Internal Medicine, Unit of Rheumatology, San Pietro Fatebenefratelli Hospital, 00189 Rome, Italy; migliore.alberto60@gmail.com; 12 Department of MeSVA, University of L’Aquila, 67100 L’Aquila, Italy; albapiroli@gmail.com; 13 Natrail Tagesklinik, 81925 Munchen, Germany; info@tapparo.de

**Keywords:** multiple chemical sensitivity (MCS), chemical intolerance, threshold limit value, environmental exposure, neurogenic inflammation

## Abstract

Multiple chemical sensitivity (MCS) is a multisystem, recurrent, environmental disorder that flares in response to different exposures (i.e., pesticides, solvents, toxic metals and molds) under the threshold limit value (TLV) calculated for age and gender in the general population. MCS is a syndrome characterized by cutaneous, allergic, gastrointestinal, rheumatological, endocrinological, cardiological and neurological signs and symptoms. We performed a systematic review of the literature to summarize the current clinical and therapeutic evidence and then oriented an eDelphi consensus. Four main research domains were identified (diagnosis, treatment, hospitalization and emergency) and discussed by 10 experts and an MCS patient. Thus, the first Italian MCS consensus had the double aim: (a) to improve MCS knowledge among healthcare workers and patients by standardizing the clinical and therapeutic management to MCS patients; and (b) to improve and shed light on MCS misconceptions not supported by evidence-based medicine (EBM).

## 1. Introduction

Multiple chemical sensitivity (MCS) is a multisystem, recurrent, environmental disorder that flares in response to different exposures (i.e., pesticides, solvents, toxic metals and molds) under the threshold limit value (TLV) calculated for age and gender in the general population [1].

From its first description in 1956 by Randolph [2], and the subsequent definition as MCS by Cullen in 1987 [3], an MCS diagnosis remains challenging for clinicians and even more so for scientists facing inclusion criteria difficulties in their studies. Remarkably, in 1999, the International Consensus on MCS [4], and then Lacour in 2005 [5], summarized a precise set of six diagnostic criteria. In the literature, MCS is described by different pseudonyms such as idiopathic environmental intolerance (IEI), environmental illness (EI), chemical intolerance (CI) or toxicant-induced loss of tolerance (TILT), which either focus on the symptoms (IEI, EI, CI) or the pathogenetic mechanisms (TILT). In the current consensus, the experts preferred the term MCS for historical and inclusive purposes.

Nowadays, MCS has an estimated prevalence of 0.5–6.5% in medically evaluated patients [6,7]; a self-reported prevalence achieves 9.0–11.2% in the general population [6,8]. In the last five years, MCS knowledge has rapidly incremented and clinical manifestations [9,10,11,12,13,14,15,16,17,18,19,20], triggers [21,22,23,24,25,26,27] and a patient category at risk [28,29,30,31] have been identified. These contribute to the understanding of the MCS pathogenesis [10,21,32,33,34,35,36,37,38,39,40,41,42,43,44,45,46,47,48] and assists in the design of dedicated MCS screening questionnaires [49,50,51,52,53,54] (Table 1).

Due to the evolution of the diagnostic criteria, the epidemiology, pathogenesis and clinical evaluation differ based on the criteria chosen. This scenario has deeply influenced the therapeutic management of MCS patients. Thus, we decided to perform a clinical and therapeutic consensus on MCS to further orient clinicians.

## 2. Materials and Methods

### 2.1. Scientific Committee

This consensus was designed and conducted by the MCS Italian Study Group that groups several experts and medical and non-medical researchers with a particular focus on MCS. The medical committee (>5 years of experience) comprises allergists/allergologists, dermatologists, rheumatologists, anesthesiologists, dentists and otorhinolaryngologists whereas the non-medical experts are biologists with at least five publications focusing on MCS in peer-reviewed international journals in the last five years (Table 2).

### 2.2. Study Design

Due to the growing body of literature on MCS and the coronavirus 2 (SARS-CoV-2) infection pandemic, experts were involved in a Delphi process shaped in a pre-Delphi with three rounds divided by 2 months each to promote an interdisciplinary discussion and a critical literature evaluation.

To define MCS, we adopted the Lacour revised criteria: (a) a chronic condition (>6 months duration) with a worsening of both quality of life and organic functions; (b) recurrent and reproducible symptoms also involving the nervous system with a characteristic hypersensitivity to odors; (c) symptoms involving the central nervous system and at least one other symptom; (d) reproducible responses to triggers at a low concentration; (e) a response to unrelated chemicals; and (f) an improvement of symptoms or even a complete resolution after the removal of the trigger [5]. Remarkably, a diagnosis can be made if the patient fulfills all six criteria.

#### 2.2.1. Pre-Delphi Exercise

A systematic review was conducted in PubMed and EMBASE using the keywords “Multiple Chemical Sensitivity”, “MCS”, “Idiopathic Environmental Intolerances”, “Environmental Illness”, “Chemical Intolerance”, “Toxicant-Induced Loss Of Tolerance”, “TILT”, “EI” and “IEI” by two MCS Study Group members (G.D. and P.D.M.P.) on 21 May 2020.

**Figure 1:**
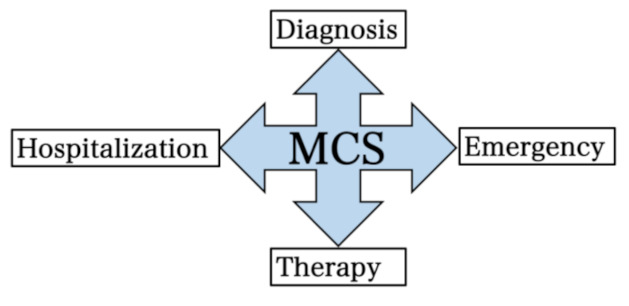
Research domains identified during the pre-Delphi. MCS: multiple chemical sensitivity.

In line with Lacour’s MCS extended criteria [5] and the clinical manifestations, we decided to create four main research domains, namely, diagnosis, therapy, hospitalization and emergency treatment (Figure 1).

#### 2.2.2. Delphi Rounds

The eDelphi exercise was designed from the feedback of the pre-Delphi exercise and proposed to 11 Italian MCS experts with a median experience of 26 (15.5–34.5) years plus a representative of the patients (Table 2). All participants rated the questions from 0 (strong disagreement) to 10 (complete agreement) and the agreement was established as ≥70%. A comment space was present after each question as well as a link to the reference used to prepare the voting statement. During all rounds, the results of the previous round were reported.

After each round, a meeting took place to discuss the potential criticisms and disagreements.

#### 2.2.3. Statistics

The continuous data were reported as a median and interquartile range and the agreement was calculated with the Fleiss Kappa coefficient (≤0, no agreement; 0.01–0.20, slight; 0.21–0.40, fair; 0.41–0.60, moderate; 0.61–0.80, substantial; 0.81–1.00, an almost perfect agreement). We reported in the results section only statements that achieved an agreement. All statistical analyses were performed by the commercial software “Statistical Package for Social Sciences” (SPSS for Windows, version 24.0, IBM, Armonk, NY, USA).

## 3. Results and Discussion

### 3.1. Pre-Delphi and eDelphi Exercises

During the pre-Delphi, the results of the systematic review were collegially discussed among six stakeholders but no agreement was reached. In every round, all 11 stakeholders and the representative of the patients were present and voted.

### 3.2. Diagnosis

**Figure 2:**
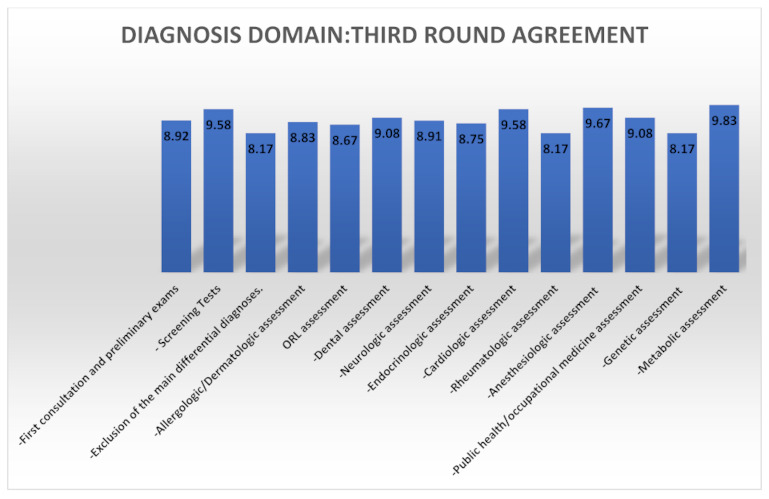
Third round agreement summary for the diagnosis domain.

The diagnostic and clinical management parameters are summarized in Table 3. The third round eDelphi agreements are summarized in Figure 2.

#### 3.2.1. First Consultation Exam to Prescribe

Medical history is of paramount importance in starting the evaluation of a potential MCS patient and may enable clinicians to set up a preliminary blood exam list. We achieved an agreement for these blood test sets according to the literature [1]:

1. Serum protein electrophoresis;
2. Ferritin serum;
3. Sodium (Na), magnesium (Mg), zinc (Zn) serum;
4. Creatine phosphokinase (CPK) serum;
5. Cholinesterase serum/plasma/erythrocyte;
6. Erythrocyte sedimentation rate (ESR);
7. C-reactive protein (CRP) serum;
8. Immunoglobulin E (Total IgE) serum;
9. Interleukin-2 receptor (sIL2r) serum;
10. Basal serum cortisol;
11. Basophil activation test on chemicals known for adverse reactions.

#### 3.2.2. Screening Tests

The Brief Environmental Exposure and Sensitivity Inventory (BREESI), validated in 2020 by Palmer et al., is a three-question, fast and easy-to-perform screening tool that screens patients with possible MCS [54] and who, consequently, must undergo the Quick Environmental Exposure and Sensitivity Inventory (QEESI©) for diagnostic purposes [50].

The QEESI©, validated in 1999 by Miller and Prihoda, was chosen to maintain international comparability and adequate accuracy [50,51]. The questionnaire has four scales of values to establish the severity of the symptoms, chemical intolerances, other intolerances and environmental impact on the health of the subject. Each scale provides a score from 0 to 10 and also includes the evaluation of the masking index or of the possible lack of awareness on the part of the interviewed subject of their intolerance and of their responses to environmental exposures [50].

In a study carried out by Miller and colleagues on 421 subjects including four exposure groups and a control group, the QEESI© had a sensitivity of 92% and a specificity of 95% in discriminating chemically sensitive people and the common population [55].

Both the BREESI and QEESI© were translated into Italian by the MCS Italian Study Group and are currently under validation.

#### 3.2.3. Main Diagnoses to Exclude

Other systemic diseases capable of fulfilling all the Lacour criteria [5] should be ruled out with particular attention paid to porphyria and macrocytosis, which have a defined set of diagnostic criteria [56,57].

#### 3.2.4. Specialist Evaluations in Patients with MCS

##### Allergologic/Dermatologic Assessment (I Level)

Despite MCS not being classified as an allergic disease, the evidence sustains an epidemiological association with an allergic disorder [58,59]. Thus, clinicians should encourage potential MCS patients to maintain a diary of symptoms. During the third eDelphi round, we achieved an agreement on the following set of tests:

1. Total immunoglobulin E (IgE) dosage and, only in the case of a clinical suspect, specific or recombinant IgE assays (Immuno Solid-Phase Allergen Chip (ISAC^®^) and in vitro multiplex allergy (i.e., Allergy Explorer-ALEX^®^ and ALEX^2^^®^) tests).
2. Patch tests are regarded as a second choice as they can cause MCS flares to the patients.
3. A lymphocyte transformation test (LTT) is optimal only for testing metal allergies and has approval/approbatory medical–legal validity only for metal allergies.

The experts agreed on the assessment and treatment of allergic and dermatologic diseases in MCS patients following the Italian Society of Allergological, Occupational and Environmental Dermatology (SIDAPA), the Italian Society of Dermatology (SIDeMaST) and the Italian Respiratory Society (SIP/IRS) guidelines.

##### Otorhinolaryngology (ORL) Assessment (I Level)

This evaluation has a pivotal role in evaluating both the functionality and reactivity of the upper airways together with the sensory pathway. The agreement was reached with this set of exams:

1. Upper airway endoscopy [11,60,61,62,63,64,65,66];
2. Olfactometry with ‘Sniffin’ Stick’ stick tests (threshold, discrimination and odor identification) and olfactory-related questionnaires [11,60,61,62,63,64,65,66];
3. An otoneurological evaluation (pure-tone audiometry and impedance examination, auditory brainstem response and otoacoustic emissions, hyperacusis and dizziness-related questionnaires, posturographic examination) [62,63,64,65,66];
4. Positron emission tomography (PET) with a pure olfactory stimulus only in selected cases with borderline or ambiguous results [11,60,61,62,63,64,65,66].

The experts agreed on the assessment and treatment of ORL diseases in MCS patients following the Italian Society of Otolaryngology and Head and Neck Surgery (SIO-CCF) guidelines.

##### Dental Assessment (I Level)

Mercury-containing dental amalgam fillings release metal ions (i.e., mercury, silver, tin, copper, gold and nickel) in the oral cavity, resulting in toxicity (i.e., neurotoxicity, immune-toxicity and hormonal dysfunction) and potential allergic reactions [67,68]. Dental prostheses and metal crowns may release gold, palladium, chromium, beryllium, cobalt and titanium. Ceramics and dental porcelain can release aluminum into the saliva and dental resin-based composite restorations can release zirconium [69].

The eDelphi results suggested that blood/serum, urine and saliva analyses should be performed to check metal toxicity [70,71,72].

Toxic Metals Screening in Blood:

1. Mercury (Hg)  whole blood.
2. Lead (Pb)      whole blood.
3. Aluminum (Al)  whole blood/serum.
4. Cadmium (Cd)  whole blood.
5. Nickel (Ni)   whole blood.

Toxic Metals Screening in Urine:

1. Mercury (Hg)    24 h urine specimens.
2. Arsenic (As)   24 h urine specimens.

The chewing-gum-stimulated saliva test represents a non-invasive and accurate method of detecting metals released in the saliva. Unfortunately, it is not currently available in Italy [73,74].

The experts agreed on the assessment and treatment of oral diseases in MCS patients following the Italian Society of Periodontology and Implantology (SIDP), the Italian Endodontic Society (SIE), the Italian Academy of Conservative Dentistry (AIC), the Italian Academy of Osseointegration (IAO), the Italian Society of Oral Pathology and Medicine (SIPMO), the Italian Academy of Endodontics (AIE), the Italian Society of Orthodontics (SIDO) and the Academy of Non-Transfusional Hemo-Components (ANTHEC) guidelines.

##### Neurological Assessment (I Level)

Despite MCS patients often displaying a normal neurological exam, environmental exposures may negatively modulate the nervous system (spatial disorientation, short-term memory loss, tinnitus, tremors, convulsions) in susceptible subjects [1]. Thus, the neurological armamentarium may also include the following clinical and instrumental tests:

1. Pupillography [75];
2. Simple and choice reaction time tasks [76];
3. Balance tests [77];
4. Visual contrast tests [78,79,80,81];
5. Visual color tests [82];
6. Tests of the perception of vibrations [77];
7. Electroencephalography (EEG) [82];
8. Single-photon emission computed tomography (SPECT) [83,84,85,86].

An assay of the serum S100B protein is recommended to evaluate the permeability of the blood–brain barrier that may be altered by MCS triggers [87]. A neuron-specific enolase (NSE) assay in serum is suggested to evaluate current or even previous mercury-related neurological signs and symptoms [88,89].

The experts agreed on the assessment and treatment of neurological diseases in MCS patients following the Italian Society of Neurology (SIN) guidelines.

##### Endocrinologic assessment (I Level)

Several metals as well as chemicals may interfere with physiology of the endocrine glands; in particular, the thyroid [90] and hypothalamic-pituitary-adrenal axis [72]. Recently, epidemiologic studies further confirmed the association between MCS and endocrinological disorders (i.e., hyposurrenalism, dysthyroidism and hyperprolactinemia) [18,50,91,92].

The experts agreed on the assessment of endocrinopathies in MCS patients following the Italian Association of Clinical Endocrinologists (AME) or the Italian Society of Endocrinology (SIE) guidelines.

##### Cardiological assessment (I Level)

MCS patients display a wide range of comorbidities including cardiovascular ones [40,93]. As well as the epidemiological associations between MCS and tachycardia, arrhythmia, a mitral valve prolapse [94] and electrocardiogram abnormalities [95,96], the cause–effect link is far from being elucidated. Rea and colleagues postulated a synergic detrimental effect of a dysregulated autonomous central nervous system with vasoconstriction due to MCS triggers in susceptible patients (i.e., diabetes and/or hypertension) [97,98].

The experts agreed on the assessment of cardiovascular disorders in MCS patients following the Italian Federation of Cardiology (IFC), the Italian Society of Cardiology (SIC) and the Italian Association for Cardiovascular Prevention and Rehabilitation (AICPR) guidelines.

##### Rheumatologic Assessment (I Level)

Several MCS patients may display an association with autoimmune diseases (Hashimoto’s thyroiditis, systemic lupus erythematosus (SLE), Sjogren’s syndrome) [38,99,100], corroborating the MCS immunological pathogenetic hypothesis [36,37,38,39].

The experts agreed on the assessment of rheumatological disorders in MCS patients following the Italian Society of Rheumatology (SIR) guidelines.

##### Anesthesiologic Assessment (I Level)

The anesthesiologic management of MCS patients remains challenging in real-life and should avoid all environmental exposures capable of triggering an MCS flare [101,102,103] (see Section 3.3 Therapy Domain).

Remarkably, MCS patients do not display an increased risk of anaphylaxis related to anesthetics (both local and systemic ones), but may experience transient postoperative symptoms currently interpreted as self-limiting flares [104]. Anesthesiologists should carefully collect the pharmacological history of MCS patients to avoid anesthetics that previously provoked anaphylaxis and/or intraoperative signs and symptoms. Pre-operative anesthetic-related allergy tests should not be performed to avoid sensitization phenomena. 

The experts agreed on the assessment of potential anesthesiologic disorders in MCS patients following the Italian Society of Anesthesia, Analgesia, Resuscitation and Intensive Care (SIAARTI) guidelines.

##### Public Health/Occupational Medicine Assessment (I Level)

Chemical, physical and biological evaluations should be performed at the working site and at home to detect any recognized MCS triggers [1] for patients with a positive QEESI©.

The experts agreed on the assessment of occupational and environmental-related disorders in MCS patients following the Italian Society of Occupational Medicine (SIML) and the Italian Society of Hygiene, Preventive Medicine and Public Health (SITI) guidelines.

##### Genetic Assessment (II Level)

Although the MCS genetic fingerprint is far from being fully elucidated, phase I and II detoxification enzymes (cytochromes P450 (CYPs), glutathione S-transferases (GSTs), N-acetyltransferases (NATs)) and antioxidant enzyme (SOD2) gene polymorphisms have been linked to MCS [105,106,107,108,109]. These polymorphisms may decrease xenobiotic catabolism and increase oxidative stress [21,105]. Thus, the gene expression is epigenetically modulated by exposure, both internal and external, leading to potential hypersensitivity and MCS [105,106,107,108,109].

Thus, the experts agreed that MCS-related polymorphism screening remains not diagnostic but only a complementary test.

##### Metabolic Assessment (II Level)

Metabolism perturbations due to or provoked by environmental exposures are currently under evaluation and the preliminary data suggest abnormalities in the detoxification metabolism (i.e., glutathione transferase, catalase, superoxide dismutase), energetic metabolism (i.e., intracellular adenosine triphosphate (ATP) in erythrocytes and platelets) and inflammatory response (pro-inflammatory serum cytokines) [43,87,105,109,110,111,112,113,114]. These promising biomarkers evaluated on serum, whole blood and peripheral blood mononuclear cells (PBMCs) are detected with methods validated only in experimental conditions and are not applicable to daily clinical practice. Thus, the experts agreed that biochemical tests should be reserved for an experimental setting.

### 3.3. Therapy Domain

**Figure 3:**
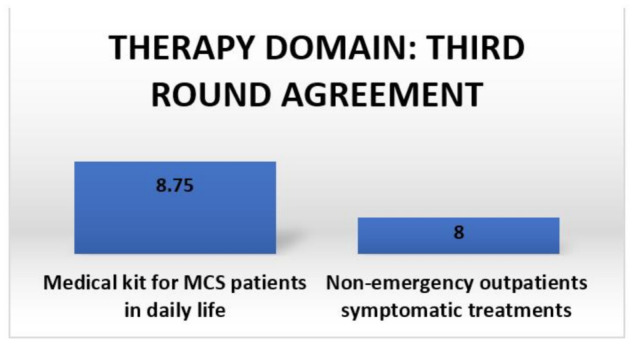
Third round agreement summary for the therapy domain.

Due to the wide range of clinical manifestations of MCS and the fragility of patients, a multidisciplinary approach (including a combination of dietetic [59] and psychological treatment strategies [110]) is mandatory. Furthermore, MCS determines disability, limiting interactions and forcing patients to purchase several medical devices to prevent MCS flares [115,116]. The agreements on this domain are summarized in Figure 3.

#### 3.3.1. Medical Kit for MCS Patients in Daily Life

During the COVID-19 pandemic, the use of medical devices (i.e., masks) has become a mandatory preventive strategy but mask choice is of paramount importance in patients with pre-existent facial dermatoses and especially in MCS patients [117,118,119]. Thus, the experts agreed to suggest this kit of medical devices for MCS patients according to their clinical profile to prevent daily flares:

1. Masks (latex-free paper face masks or cotton masks and filters and/or masks with a high-efficiency particulate absorbing filter (HEPA) and activated carbon filters).
2. Air purifiers (portable household air in metal with HEPA filters with activated carbon and a percentage of rubber gaskets < 3% and relative filters and/or air purifiers for cars in metal with HEPA filters with activated carbon and a percentage of rubber gaskets < 3% and relative filters. Air filters should be supplied with an oxygen tank and a glass oxygen bubbler and be phthalate-free and flexible with an oxygen tube with a ceramic mask and latex-free glasses).
3. Water purifiers (an active carbon water purifier with a percentage of rubber gaskets < 3%).

All devices should have the CE (Conformité Européenne) mark and masks may guarantee SARS-CoV-2 protection at least equal to surgical masks. If the mask does not display surgical mask protection characteristics, it should be supplemented with a surgical mask on top to prevent a SARS-CoV-2 infection.

#### 3.3.2. Symptomatic Treatments for Non-Emergency Outpatients 

Due to the fragility of MCS patients and frequent allergies, a careful evaluation of prescribed drugs (i.e., avoid colored tablets and assess non-active components) and the assessment of medical devices (i.e., avoid plastic and glass ones) are mandatory [1].

Pharmacological therapy must start at half the dosage and carefully increase until the adequate dose is reached to ensure the tolerability of the MCS patient to the drug.

The oximetry should be carefully evaluated in MCS patients and also during MCS flares before delivering any oxygen therapy. An intranasal administration of hyaluronic acid may alleviate olfactory discomfort [63].

### 3.4. Hospitalization Domain

**Figure 4:**
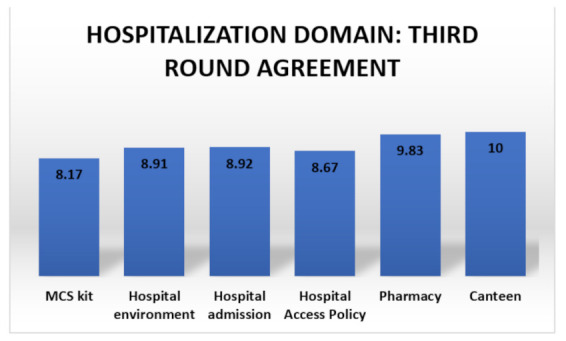
Third round agreement summary for the hospitalization domain.

Healthcare workers should undergo dedicated MCS training to safely manage MCS patients at every step of hospitalization (i.e., environment, admission, access policy, pharmacy and canteen) [1,115]. The agreements on this domain are summarized in Figure 4.

The eDelphi experts agreed that an MCS kit marked with a distinct color should be established in hospitals to maximize the therapeutic management efficacy and it should include:

1. Latex-free surgical gloves;
2. Cleaning products without perfumes and hydrogen peroxide;
3. Hydrogen peroxide for disinfection;
4. 5% dextrose (glucose-intravenous) in a 1000 cc 0.9% NaCl glass drip;
5. Porcelain oxygen mask;
6. Phthalate-free, flexible oxygen tube;
7. Latex-free glasses;
8. Inverted sugar solution in a 1000 cc 0.9% NaCl glass drip;
9. Sodium bicarbonate solution in glass vials (500 cc);
10. Intravenous administration kit in glass;
11. Sheets, pillowcases, tablecloths, sterile cotton towels, washed cotton pillows with non-perfumed detergents and without softener (not dry-cleaned);
12. Disposable cotton tunics washed with fragrance-free detergents;
13. Disposable headgear, shoe covers and tunics;
14. Latex-free paper plasters;
15. Intravenous butterfly valve;
16. Velcro tourniquet/cuff sphygmomanometer;
17. Fragrance-free soap for healthcare workers in contact with MCS patients;
18. Latex-free paper masks for healthcare workers in contact with MCS patients;
19. A 0.9% NaCl 1000 cc solution drip in glass.

#### 3.4.1. Hospital Environment

The eDelphi experts found a substantial agreement between the following statements. MCS patients should be conducted in the ambulatory, if possible, without crossing waiting rooms and from a different entrance. Furthermore, the MCS ambulatory should be far from sterile processing facilities, laundries, waste rooms or any sources of internal and external potential MCS triggers. Solvents, pesticides and herbicides or any other potentially toxic chemical agent dispersions should be avoided in the external area adjacent to the MCS ambulatory. The ambulatory should be covered by unpolished natural stoneware majolica on the floor and walls to decrease the cleaning time and prevent chemical absorbance. Natural light is preferred to artificial light.

#### 3.4.2. Hospital Admission

The experts agreed that healthcare workers should:

1. Arrange MCS patients in a private room marked with a dedicated color (i.e., the MCS kit) with advice prohibiting the access of any person with perfumes;
2. Prioritize the arrangement in ventilated rooms far from sources of MCS-recognized triggers (i.e., streets);
3. Decontaminate the room in advance (>6 h before the admission);
4. Clean the room with water, bicarbonate and fragrance-free detergents;
5. Use sheets, pillowcases and 100% cotton towels;
6. Mark in the clinical history any allergies, previous drug reactions and tolerated drugs with particular attention paid to antibiotics, anesthetics and disease-modifying antirheumatic drugs (DMARDs);
7. Pre-alert the hospital pharmacy, healthcare workers and the canteen service;
8. Provide water only in glass bottles with glass cups.

#### 3.4.3. Hospital Access Policy

The experts agreed that every person who accesses the MCS room should:

1. Avoid any perfumes, spray or hair products;
2. Wash hands with fragrance-free soap or white soap;
3. Change their clothes in a dedicated pre-entrance vestibule or a locker room, disinfected and cleaned as an MCS room;
4. Have a dedicated MCS kit that contains shirts, gloves (powder-free vinyl or nitrile), latex-free and phthalate-free oxygen tubes and a latex-free oxygen mask.

#### 3.4.4. Pharmacy

The experts agreed on these statements about the hospital pharmacy facility:

1. Use only glass bottles for intravenous solutions;
2. Do not replace tolerated drugs with generic pharmaceutical products or even with biosimilars (for target therapies);
3. Galenic preparations are preferred to packaged drugs due to their lower concentration of preservatives;
4. Carefully monitor the drug intake of MCS patients.

#### 3.4.5. Canteen

The eDelphi experts agreed on these statements about the hospital canteen:

1. Pre-alert the canteen;
2. Refer previous food reactions to the canteen;
3. Do not cook in aluminum or copper pots;
4. Use only glasses, iron cutlery and glass transparent plates (no colored glassware);
5. Report any adverse events in the medical history regarding food or beverages.

### 3.5. Emergency Domain

**Figure 5:**
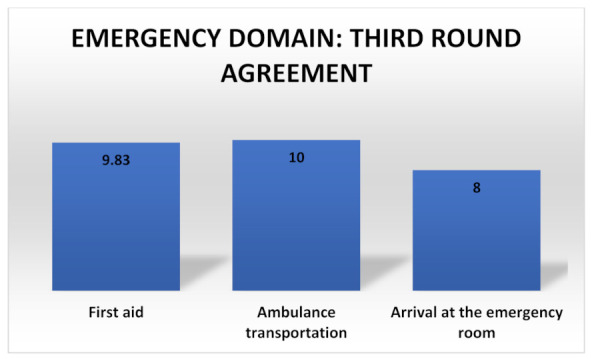
Agreements summary for the emergency domain.

MCS patients display a higher risk than the general population of being hospitalized [1] so first aid, ambulance transportation and the arrival at the emergency room should be standardized. The MCS management during an emergency should be integrated in both volunteer and professional healthcare workers. The agreements on this domain are summarized in Figure 5.

#### 3.5.1. First Aid

During the eDelphi, the experts agreed on the creation of an Emergency Kit for MCS that includes:

1. Latex-free and powder-free gloves;
2. Latex-free materials for healthcare workers;
3. Latex-free oxygen glasses for the patient;
4. Hydrogen peroxide solution to decontaminate;
5. Glass drip bottles;
6. Aluminum roll to seal off any parts of medical equipment (i.e., tubes, rubber gaskets) potentially contaminated by MCS-recognized triggers;
7. Ice gown;
8. Headgear;
9. Disposable paper shoe covers.

#### 3.5.2. Ambulance Transportation

Experts suggest the following tips for ambulance transportation:

1. Avoid environmental deodorants;
2. Healthcare workers should avoid smoke, perfumes, hair gel or deodorants 6 h before an ambulance shift;
3. Use the emergency kit for MCS.

#### 3.5.3. Arrival at the Emergency Room

The eDelphi experts agreed on the following suggestions for MCS patients arriving at the emergency room:

1. Isolate MCS patients from the other patients and place visitors into a separate room;
2. Decontaminate the separate room and remove all potential MCS triggers (i.e., solvents, rubber parts);
3. Assign a priority code to the MCS patients;
4. Use the MCS kit.

## 4. Conclusions

This is the first Italian consensus and one of the few consensuses worldwide on MCS that aims to orient daily practice and improve the quality of delivered treatments to MCS patients. The MCS evidence in the literature remains scant so future studies should evaluate in deeper detail the clinical, epidemiological and therapeutic unmet needs.

## Figures and Tables

**Table 1:** Clinical and pathogenetic overview of MCS characteristics.

Pathogenetic Hypotheses	Clinical Manifestations *	Screening Questionnaires	Subjects at Risk	Triggers *
Limbic dysfunction [34,35,36,37]	Neurological disorders [1,12,13,14,15,16,17]: headache, migraine, trigeminal neuralgia, convulsions, attention deficit disorder, neurocognitive deficits, hyperacusis, insomnia, myalgic encephalomyelitis	Environmental Exposure and Sensitivity Intolerance (EESI) [49]	Industrial workers acutely or chronically exposed to recognized triggers [30]	Organic solvents and related compounds [23]
Immune disorders [36,37,38]	ORL disorders [1]: sinusitis, polyps, non-allergic rhinitis with eosinophilic syndrome, tinnitus, recurrent otitis, allergic rhinitis	Quick Environmental Exposure and Sensitivity Inventory (QEESI) [50]	Other workers exposed to recognized triggers (farmers, hairdressers, radiologists, anesthesiologists) [30]	Insecticides, pesticides, herbicides [23]
Biochemical mechanisms [40,41,42,43]	Cardiovascular disorders [1]: arrhythmia, tachycardia, hypotension, hypertension, Raynaud’s phenomenon, lipothymia	Huppe Questionnaire [51]	Office workers [30]	Different gases (i.e., hydrogen sulfide (H_2_S) or carbon monoxide (CO) [23]
Neurogenic inflammation [39]	Respiratory disorders [1]: asthma, tracheitis, bronchospasms, chronic tonsillitis, hyper-reactive airway syndrome, toluene diisocyanate hypersensitivity	Chemical Sensitivity Scale for Sensory Hyper-Reactivity (CSS-SHR) [52]	Residents in contaminated areas [30]	Metals (i.e., mercury) [23,30]
Neurophysiological and respiratory mechanisms [44,45]	Gastroenterological disorders [1]: irritable colon, colitis, gastroesophageal reflux (GERD), celiac disease, gluten sensitivity, food intolerances, food allergies	German Questionnaire on Chemical and Environmental Sensitivity (CGES) [53]	Gulf War veterans [30]	Molds and mycotoxins [24,25,26,27,28]
Vascular dysfunction [46]	Rheumatological disorders: fibromyalgia, carpal tunnel syndrome, dysfunction of the temporomandibular joint (TMJ), arthritis, connective tissue disease, systemic lupus erythematosus (SLE) [1]	Brief Environmental Exposure and Sensitivity Inventory (BREESI) [54]	Silicon or prosthesis implants carriers [10,31]	Xenobiotics in foods and beverages (i.e., sulfites) [29]
Psychiatric disorders [47,48]	Dermatological/allergic disorders [1]: eczema, systemic dermatitis, rash, urticaria/angioedema, photosensitivity, skin photosensitivity, dermographism		Patients born by Caesarean section [33]	Combusted products (diesel exhaust, tobacco, wood) [29]
N-Methyl-D-aspartic acid or N-Methyl-D-aspartate (NMDA) sensitization and stimulation by reactive oxygen species and peroxynitrite [21]	Endocrinological disorders [1,18]: diabetes, dysthyroidism, adrenal gland disorders, pituitary disorders			Other substances (natural psoralens, terpenes) [29]
	Psychological/psychiatric disorders: anxiety, depression, manic depression, bipolar disorder, mood swings, panic attacks			
	Others [19,20]: Chronic Fatigue Syndrome, Gulf War Syndrome, Sick Building Syndrome			

* Reported in the literature; the exact causative mechanism remains elusive.

**Table 2:** Characteristics of the members in the pre-Delphi and Delphi consensus exercises.

Demographics and Characteristics	Pre-Delphi Exercise (N = 7)	Delphi Rounds (N = 12)
Dermatologists, N (%)	1 (14.3)	1 (8.3)
Dentists, N (%)	1 (14.3)	2 (16.6)
Otorhinolaryngologist, N (%)	1 (14.3)	1 (8.3)
Anesthesiologists, N (%)	-	1 (8.3)
Allergists/Allergologists, N (%)	1 (14.3)	1 (8.3)
Rheumatologists	-	1 (8.3)
Alternative Medicine Doctors	-	1 (8.3)
Biologists, N (%)	2 (28.6)	2 (16.6)
Representatives of Patients, N (%)	1 (14.3)	1 (8.3)
Male, N (%)	5 (71.4)	9 (75.0)
Age, Median (IQR), Years	52 (50–57.5)	55 (47–59)
Clinical/Research Experience, Median (IQR), Years	25.5 (21.3–27.5)	26 (15.5–34.5)
Academic Experience, N (%) *	5 (71.4)	5 (41.7)
Hospital or Private Practice Experience, N (%)	4 (57.1)	9 (75.0)
Both, N (%)	6 (85.7)	11 (91.7)

IQR: Interquartile range. * Among the experts, five had an academic position: 1 Postdoctoral Fellow (G.D.), 1 Assistant Professor (A.M.) and 3 Associate Professors (A.M, D.C. and P.D.M.P.).

**Table 3:** Diagnostic and clinical parameters of MCS patients.

Screening and Diagnosis (Level 0)	1st Level Assessments	2nd Level Assessments
− First consultation and preliminary exams − Screening tests − Exclusion of the main differential diagnoses	− Allergologic/dermatologic assessment − ORL assessment * − Dental assessment − Neurologic assessment − Endocrinologic assessment − Cardiologic assessment − Rheumatologic assessment − Anesthesiologic assessment − Public health/occupational medicine assessment	− Genetic assessment − Metabolic assessment

* ORL: Otorhinolaryngology.

## Data Availability

No new data were created or analyzed in this study. Data sharing is not applicable to this article.

## References

[1] Azuma K., Uchiyama I., Tanigawa M., Bamba I., Azuma M., Takano H., Yoshikawa T., Sakabe K. (2019). Chemical intolerance: Involvement of brain function and networks after exposure to extrinsic stimuli perceived as hazardous. Environ. Health Prev. Med..

[2] Randolph T.G. (1961). Human ecology and susceptibility to the chemical environment. Ann. Allergy.

[3] Cullen M.R. (1987). The worker with multiple chemical sensitivities: An overview. Occup. Med..

[4] Bartha L., Baumzweiger W., Buscher D.S., Callender T., Dahl K.A., Davidoff A., Donnay A., Edelson S.B., Elson B.D., Elliot E. (1999). Multiple chemical sensitivity: A 1999 consensus. Arch Environ. Health.

[5] Lacour M., Zunder T., Schmidtke K., Vaith P., Scheidt C. (2005). Multiple chemical sensitivity syndrome: Suggestions for an extension of the US. MCS case definition. Int. J. Hyg. Environ. Health.

[6] Hausteiner C., Bornschein S., Hansen J., Zilker T., Forstl H. (2005). Self-reported chemical sensitivity in Germany: A population-based survey. Int. J. Hyg. Environ. Health.

[7] Pigatto P.D., Guzzi G. (2019). Prevalence and risk factors for multiple chemical sensitivity in Australia. Prev. Med. Rep..

[8] Caress S.M., Steinemann A.C. (2005). A national population study of the prevalence of multiple chemical sensitivity. Arch Environ. Health.

[9] Miller C.S. (2001). Toxicant-induced loss of tolerance. Addiction.

[10] Bell I.R., Hardin E.E., Baldwin C.M., Schwartz G.E. (1995). Increased limbic system symptomatology and sensitizability of young adults with chemical and noise sensitivities. Environ. Res..

[11] Viziano A., Micarelli A., Alessandrini M. (2017). Noise sensitivity and hyperacusis in patients affected by multiple chemical sensitivity. Int. Arch Occup. Environ. Health.

[12] Heinonen-Guzejev M., Koskenvuo M., Mussalo-Rauhamaa H., Vuorinen H.S., Heikkilä K., Kaprio J. (2012). Noise sensitivity and multiple chemical sensitivity scales: Properties in a population based epidemiological study. Noise Health.

[13] Emmett E.A. (1976). Parosmia and hyposmia induced by solvent exposure. Br. J. Ind. Med..

[14] Doty R.L., Deems D.A., Frye R.E., Pelberg R., Shapiro A. (1988). Olfactory sensitivity, nasal resistance, and autonomic function in patients with multiple chemical sensitivities. Arch. Otolaryngol. Head Neck Surg..

[15] Fernandez M., Schwartz G.E., Bell I.R. (1999). Subjective ratings of odorants by women with chemical sensitivity. Toxicol. Ind. Health.

[16] Mazzatenta A., Pokorski M., Di Giulio C. (2015). Real time analysis of volatile organic compounds (VOCs) in centenarians. Respir. Physiol. Neurobiol..

[17] Yunus M.B. (2008). Central sensitivity syndromes: A new paradigm and group nosology for fibromyalgia and overlapping conditions, and the related issue of disease versus illness. Semin. Arthritis Rheum..

[18] Pigatto P.D., Minoia C., Ronchi A., Brambilla L., Ferruci S.M., Spadari F., Passoni M., Somalvico F., Bombeccari G.P., Guzzi G. (2013). Allergological and toxicological aspects in a multiple chemical sensitivity cohort. Oxid. Med. Cell Longev..

[19] Gibson P.R., Lindberg A. (2011). Physicians’ perceptions and practices regarding patient reports of multiple chemical sensitivity. ISRN Nurs..

[20] Wiesmuller G.A., Hornberg C. (2017). [Environmental medical syndromes]. Bundesgesundheitsblatt Gesundh. Gesundh..

[21] Pall M.L. (2003). Elevated nitric oxide/peroxynitrite theory of multiple chemical sensitivity: Central role of N-methyl-D-aspartate receptors in the sensitivity mechanism. Environ. Health Perspect..

[22] Vojdani A., Thrasher J.D., Madison R.A., Gray M.R., Heuser G., Campbell A.W. (2004). Antibodies to molds and satratoxin in individuals exposed in water-damaged buildings. Arch. Environ. Health.

[23] Rea W.J. (2018). A Large Case-series of Successful Treatment of Patients Exposed to Mold and Mycotoxin. Clin. Ther..

[24] Lieberman A., Rea W., Curtis L. (2006). Adverse health effects of indoor mold exposure. J. Allergy Clin. Immunol..

[25] Hyvönen S., Poussa T., Lohi J., Tuuminen T. (2021). High prevalence of neurological sequelae and multiple chemical sensitivity among occupants of a Finnish police station damaged by dampness microbiota. Arch. Environ. Occup. Health.

[26] Hyvönen S., Lohi J., Tuuminen T. (2020). Moist and Mold Exposure is Associated with High Prevalence of Neurological Symptoms and MCS in a Finnish Hospital Workers Cohort. Saf. Health Work.

[27] Meggs W.J. (2017). The Role of Neurogenic Inflammation in Chemical Sensitivity. Ecopsychology.

[28] Miller C.S., Mitzel H.C. (1995). Chemical sensitivity attributed to pesticide exposure versus remodeling. Arch. Environ. Health.

[29] Stejskal V.D., Danersund A., Lindvall A., Hudecek R., Nordman V., Yaqob A., Mayer W., Bieger W., Lindh U. (1999). Metal-specific lymphocytes: Biomarkers of sensitivity in man. NeuroEndocrinol. Lett..

[30] Sterzl I., Prochazkova J., Hrda P., Bartova J., Matucha P., Stejskal V.D.M. (1999). Mercury and nickel allergy: Risk factors in fatigue and autoimmunity. NeuroEndocrinol. Lett..

[31] Watai K., Fukutomi Y., Hayashi H., Kamide Y., Sekiya K., Taniguchi M. (2018). Epidemiological association between multiple chemical sensitivity and birth by caesarean section: A nationwide case-control study. Environ. Health.

[32] Rainville P., Bushnell M.C., Duncan G.H. (2001). Representation of acute and persistent pain in the human CNS: Potential implications for chemical intolerance. Ann. N.Y. Acad. Sci..

[33] Bell I.R., Miller C.S., Schwartz G.E. (1992). An olfactory-limbic model of multiple chemical sensitivity syndrome: Possible relationships to kindling and affective spectrum disorders. Biol. Psychiatry.

[34] Bell I.R., Schwartz G.E., Peterson J.M., Amend D. (1993). Self-reported illness from chemical odors in young adults without clinical syndromes or occupational exposures. Arch. Environ. Health.

[35] Bell I.R., Schwartz G.E., Peterson J.M., Amend D., Stini W.A. (1993). Possible time-dependent sensitization to xenobiotics: Self-reported illness from chemical odors, foods, and opiate drugs in an older adult population. Arch. Environ. Health.

[36] Albright J.F., Goldstein R.A. (1992). Is there evidence of an immunologic basis for multiple chemical sensitivity?. Toxicol. Ind. Health.

[37] Broughton A., Thrasher J.D., Gard Z. (1988). Immunological evaluation of four arc welders exposed to fumes from ignited polyurethane (isocyanate) foam: Antibodies and immune profiles. Am. J. Ind. Med..

[38] Levin A.S., Byers V.S. (1992). Multiple chemical sensitivities: A practicing clinician’s point of view. Clinical and immunologic research findings. Toxicol. Ind. Health.

[39] Ziem G.E., Davidoff L.L. (1992). Illness from chemical "odors": Is the health significance understood?. Arch Environ. Health.

[40] Galland L. (1987). Biochemical abnormalities in patients with multiple chemical sensitivities. Occup. Med..

[41] Johnson A., Rea W.J. Review of 200 cases in the environmental control unit. Proceedings of the 7th International Symposium on Man and His Environment in Health and Disease.

[42] Levine S.A., Reinhardt J.H. (1983). Biochemical-pathology initiated by free radicals, oxidant chemicals, and therapeutic drugs in the etiology of chemical hypersensitivity disease. Orthomol. Psych..

[43] De Luca C., Scordo M.G., Cesareo E., Pastore S., Mariani S., Maiani G., Stancato A., Loreti B., Valacchi G., Lubrano C. (2010). Biological definition of multiple chemical sensitivity from redox state and cytokine profiling and not from polymorphisms of xenobiotic-metabolizing enzymes. Toxicol. Appl. Pharmacol..

[44] Mazzatenta A., Di Giulio C., Pokorski M. (2013). Pathologies currently identified by exhaled biomarkers. Respir. Physiol. Neurobiol..

[45] Mazzatenta A., Pokorski M., Cozzutto S., Barbieri P., Veratti V., Di Giulio C. (2013). Non-invasive assessment of exhaled breath pattern in patients with multiple chemical sensibility disorder. Adv. Exp. Med. Biol..

[46] Fisherman E.W., Cohen G. (1973). Chemical intolerance to butylated-hydroxyanisole (BHA) and butylated-hydroxytoluene (BHT) and vascular response as an indicator and monitor of drug intolerance. Ann. Allergy..

[47] Black D.W. (2000). The relationship of mental disorders and idiopathic environmental intolerance. Occup. Med..

[48] Staudenmayer H., Binkley K.E., Leznoff A., Phillips S. (2004). Idiopathic environmental intolerance: Part 1: A causation analysis applying Bradford Hill’s criteria to the toxicogenic theory. Toxicol. Rev..

[49] Miller C.S., Prihoda T.J. (1999). The environmental exposure and sensitivity inventory (EESI): A standardized approach for measuring chemical intolerances for research and clinical applications. Toxicol. Ind. Health.

[50] Hojo S., Kumano H., Yoshino H., Kakuta K., Ishikawa S. (2003). Application of Quick Environment Exposure Sensitivity Inventory (QEESI) for Japanese population: Study of reliability and validity of the questionnaire. Toxicol. Ind. Health.

[51] Eis D., Helm D., Mühlinghaus T., Birkner N., Dietel A., Eikmann T., Gieler U., Herr C., Lacour M., Nowak D. (2008). The German Multicentre study on multiple chemical sensitivity (MCS). Int. J. Hyg. Environ. Health.

[52] Andersson L., Johansson A., Millqvist E., Nordin S., Bende M. (2008). Prevalence and risk factors for chemical sensitivity and sensory hyperreactivity in teenagers. Int. J. Hyg. Environ. Health.

[53] Österberg K., Persson R., Karlson B., Carlsson Eek F., Orbaek P. (2007). Personality, mental distress, and subjective health complaints among persons with environmental annoyance. Hum. Exp. Toxicol..

[54] Palmer R.F., Jaén C.R., Perales R.B., Rincon R., Forster J.N., Miller C.S. (2020). Three questions for identifying chemically intolerant individuals in clinical and epidemiological populations: The Brief Environmental Exposure and Sensitivity Inventory (BREESI). PLoS ONE.

[55] Miller C.S., Prihoda T.J. (1999). A controlled comparison of symptoms and chemical intolerances reported by Gulf War veterans, implant recipients and persons with multiple chemical sensitivity. Toxicol. Ind. Health.

[56] Stölzel U., Doss M.O., Schuppan D. (2019). Clinical Guide and Update on Porphyrias. Gastroenterology.

[57] Pardanani A. (2019). Systemic mastocytosis in adults: 2019 update on diagnosis, risk stratification and management. Am. J. Hematol..

[58] Pigatto P.D., Guzzi G. (2019). Contact allergy to metals and multiple chemical sensitivity. Contact Dermat..

[59] Pigatto P.D., Rossi V., Guzzi G. (2020). Dietary factors and endocrine consequences of multiple chemical sensitivity. Endocrinol. Diabetes Nutr..

[60] Viziano A., Micarelli A., Pasquantonio G., Della-Morte D., Alessandrini M. (2018). Perspectives on multisensory perception disruption in idiopathic environmental intolerance: A systematic review. Int. Arch. Occup. Environ. Health.

[61] Alessandrini M., Micarelli A., Chiaravalloti A., Bruno E., Danieli R., Pierantozzi M., Genovesi G., Öberg J., Pagani M., Schillaci O. (2016). Involvement of Subcortical Brain Structures During Olfactory Stimulation in Multiple Chemical Sensitivity. Brain Topogr..

[62] Chiaravalloti A., Pagani M., Micarelli A., Di Pietro B., Genovesi G., Alessandrini M., Schillaci O. (2015). Cortical activity during olfactory stimulation in multiple chemical sensitivity: A (18)F-FDG PET/CT study. Eur. J. Nucl. Med. Mol. Imaging.

[63] Alessandrini M., Micarelli A., Bruno E., Ottaviani F., Conetta M., Cormano A., Genovesi G. (2013). Intranasal administration of hyaluronan as a further resource in olfactory performance in multiple chemical sensitivity syndrome. Int. J. Immunopathol. Pharmacol..

[64] Alessandrini M., Micarelli A., Chiaravalloti A., Candidi M., Bruno E., Di Pietro B., Schillaci O., Pagani M. (2014). Cortico-subcortical metabolic correlates of olfactory processing in healthy resting subjects. Sci. Rep..

[65] Micarelli A., Viziano A., Bruno E., Micarelli E., Alessandrini M. (2016). Vestibular impairment in Multiple Chemical Sensitivity: Component analysis findings. J. Vestib. Res..

[66] Micarelli A., Viziano A., Genovesi G., Bruno E., Ottaviani F., Alessandrini M. (2016). Lack of contralateral suppression in transient-evoked otoacoustic emissions in multiple chemical sensitivity: A clinical correlation study. Noise Health.

[67] Guzzi G., Grandi M., Guzzi G., Cattaneo C., Calza S., Minoia C., Ronchi A., Gatti A., Severi G. (2006). Dental amalgam and mercury levels in autopsy tissues: Food for thought. Am. J. Forensic Med. Pathol..

[68] Daunderer M. (1992). Handbuch der Amalgam-Vergiftung.

[69] Pigatto P.D., Ferrucci S., Brambilla L. Toxic metals screening in MCS patients. Proceedings of the 16th Euro-Global Summit on Toxicology and Applied Pharmacology.

[70] Guzzi G., Pigatto P.D., Legori A., Ferrucci S., Brambilla L. (2018). Multiple sensitization to metals in MCS. Contact Dermat..

[71] Guzzi G., Ronchi A., Barbaro M., Spadari F., Bombeccari G., Brambilla L., Ferrucci S., Pigatto P.D. (2016). Multiple chemical sensitivity and toxic metals. Toxicol. Lett..

[72] Guzzi G., Pigatto P.D., Ronchi A., Dolcetta D., Brambilla L., Ferrucci S., Passoni M. (2018). Exposure to metals, multiple chemical sensitivity, and neurogenic inflammation. J. Clin. Toxicol..

[73] Pigatto P., Arancio L., Guzzi G., Severi G. (2005). Metals from amalgam in saliva: Association with lichenoid lesions, leukoplakia, burning mouth syndrome. Toxicol. Lett..

[74] Pigatto P.D., Minoia C., Ronchi A., Guzzi G. (2009). Mercury in saliva: Immunotoxic and allergenic metal. All. Asthma. Proc..

[75] Shirakawa S.R., Ishikawa S., Johnson A.R. (1991). Evaluation of the autonomic nervous system response by pupillographical study in the chemically sensitive patient. Environ. Med..

[76] Weiss B. (1997). Experimental strategies for research on multiple chemical sensitivity. Environ. Health Perspect..

[77] Kilburn K.H. (1998). Chemical Brain Injury (Environmental Health).

[78] Anger W.K., Letz R., Chrislip D.W., Frumkin H., Hudnell K., Russo J.M., Chappell W., Hutchinson L. (1994). Neurobehavioral test methods for environmental health studies of adults. Neurotoxicol. Teratol..

[79] Hudnell H.K., Benignus V.A. (1989). Carbon monoxide exposure and human visual detection thresholds. Neurotoxicol. Teratol..

[80] Hudnell H.K., Boyes W.K., Otto D.A., Frumkin H., Hudnell K., Russo J.M., Chappell W., Hutchinson L. (1996). Battery of neurobehavioral tests recommended to ATSDR: Solvent-induced deficits in microelectronic workers. Toxicol. Ind. Health..

[81] Hudnell H.K., Otto D.A., House D.E. (1996). The influence of vision on computerized neurobehavioral test scores: A proposal for improving test protocols. Neurotoxicol. Teratol..

[82] Seppalainen A.M., Raitta C., Huuskonen M.S. (1979). n-Hexane-induced changes in visual evoked potentials and electroretinograms of industrial workers. Electroencephalogr. Clin. Neurophysiol..

[83] Callender T.J., Morrow L., Subramanian K. (1994). Evaluation of chronic neurological sequelae after acute pesticide exposure using SPECT brain scans. J. Toxicol. Environ. Health.

[84] Callender T.J., Morrow L., Subramanian K., Duhon D., Ristovv M. (1993). Three-dimensional brain metabolic imaging in patients with toxic encephalopathy. Environ. Res..

[85] Heuser G., Mena I., Alamos F. (1994). NeuroSPECT findings in patients exposed to neurotoxic chemicals. Toxicol. Ind. Health.

[86] Hillert L., Musabasic V., Berglund H., Ciumas C., Savic I. (2006). Odor processing in multiple chemical sensitivity. Hum. Brain Mapp..

[87] Belpomme D., Campagnac C., Irigaray P. (2015). Reliable disease biomarkers characterizing and identifying electrohypersensitivity and multiple chemical sensitivity as two etiopathogenic aspects of a unique pathological disorder. Rev. Environ. Health.

[88] Guzzi G., Ronchi A., Bolengo I., Pontillo M., Soldini L., Soldarini A., Pigatto P.D. (2015). NSE: Marker of the Clinical Toxicity of Mercury. Toxicol. Lett..

[89] Pigatto P., Ronchi A., Guzzi G. (2014). NSE as a biomarker of mercury exposure. Clin. Toxicol..

[90] Wade M.G., Parent S., Finnson K.W., Foster W., Younglai E., McMahon A., Cyr D.G., Hughes C. (2002). Thyroid toxicity due to subchronic exposure to a complex mixture of 16 organochlorines, lead, and cadmium. Toxicol. Sci..

[91] Baines C.J., McKeown-Eyssen G.E., Riley N., Cole D.E., Marshall L., Loescher B., Jazmaji V. (2004). Case-control study of multiple chemical sensitivity, comparing haematology, biochemistry, vitamins and serum volatile organic compound measures. Occup. Med..

[92] Bell I.R., Bootzin R.R., Davis T.P., Hau V., Ritenbaugh C., Johnson K.A., Schwartz G.E. (1996). Time-dependent sensitization of plasma beta-endorphin in community elderly with self-reported environmental chemical odor intolerance. Biol. Psychiatry.

[93] Haumann K., Kiesswetter E., van Thriel C., Blaszkewicz M., Golka K., Seeber A. (2003). Breathing and heart rate during experimental solvent exposure of young adults with self-reported multiple chemical sensitivity (sMCS). Neurotoxicology.

[94] Ziem G., McTamney J. (1997). Profile of patients with chemical injury and sensitivity. Environ. Health Perspect..

[95] Bell I.R., Schwartz G.E., Hardin E.E., Baldwin C.M., Kline J.P. (1998). Differential resting quantitative electroencephalographic alpha patterns in women with environmental chemical intolerance, depressives, and normals. Biol. Psychiatry.

[96] Baldwin C.M., Bell I.R. (1998). Increased cardiopulmonary disease risk in a community-based sample with chemical odor intolerance: Implications for women’s health and health-care utilization. Arch. Environ. Health.

[97] Rea W.J. (1976). Environmentally triggered thrombophlebitis. Ann. Allergy.

[98] Rea W.J. (1977). Environmentally triggered small vessel vasculitis. Ann. Allergy.

[99] Slotkoff A.T., Radulovic D.A., Clauw D.J. (1997). The relationship between fibromyalgia and the multiple chemical sensitivity syndrome. Scand. J. Rheumatol..

[100] Migliore A., Bizzi E., Massafra U., Capuano A., Martin Martin L.S. (2007). Multiple chemical sensitivity syndrome in Sjogren’s syndrome patients: Casual association or related diseases?. Arch. Environ. Occup. Health.

[101] Gibson P.R., Elms A.N., Ruding L.A. (2003). Perceived treatment efficacy for conventional and alternative therapies reported by persons with multiple chemical sensitivity. Environ Health Perspect..

[102] Skovbjerg S., Brorson S., Rasmussen A., Johansen J.D., Elberling J. (2009). Impact of self-reported multiple chemical sensitivity on everyday life: A qualitative study. Scand. J. Public Health.

[103] Piroli A., Ciccozzi A., Petrucci E., Paladini A., Marsili I., Panella L., Santucci C., Coaccioli S., Marinangeli F. (2013). Anaesthesia management in patients with multiple chemical sensitivity syndrome. Int. J. Immunopathol. Pharmacol..

[104] Fisher M.M., Rose M. (2008). Anaesthesia for patients with idiopathic environmental intolerance and chronic fatigue syndrome. Br. J. Anaesth..

[105] McKeown-Eyssen G., Baines C., Cole D.E., Riley N., Tyndale R.F., Marshall L., Jazmaji V. (2004). Case-control study of genotypes in multiple chemical sensitivity: CYP2D6, NAT1, NAT2, PON1, PON2 and MTHFR. Int. J. Epidemiol..

[106] Berg N.D., Rasmussen H.B., Linneberg A., Brasch-Andersen C., Fenger M., Dirksen A., Vesterhauge S., Werge T., Elberling J. (2010). Genetic susceptibility factors for multiple chemical sensitivity revisited. Int. J. Hyg. Environ. Health.

[107] Fujimori S., Hiura M., Yi C.X., Xi L., Katoh T. (2012). Factors in genetic susceptibility in a chemical sensitive population using QEESI. Environ. Health Prev. Med..

[108] Cui X., Lu X., Hiura M., Oda M., Miyazaki W., Katoh T. (2013). Evaluation of genetic polymorphisms in patients with multiple chemical sensitivity. PLoS ONE.

[109] Caccamo D., Cesareo E., Mariani S., Raskovic D., Ientile R., Currò M., Korkina L., De Luca C. (2013). Xenobiotic sensor- and metabolism-related gene variants in environmental sensitivity-related illnesses: A survey on the Italian population. Oxid. Med. Cell Longev..

[110] Micarelli A., Cormano A., Caccamo D., Alessandrini M. (2019). Olfactory-Related Quality of Life in Multiple Chemical Sensitivity: A Genetic-Acquired Factors Model. Int. J. Mol. Sci..

[111] De Luca C., Gugliandolo A., Calabrò C., Currò M., Ientile R., Raskovic D., Korkina L., Caccamo D. (2015). Role of polymorphisms of inducible nitric oxide synthase and endothelial nitric oxide synthase in idiopathic environmental intolerances. Mediat. Inflamm..

[112] Gugliandolo A., Gangemi C., Calabrò C., Vecchio M., Di Mauro D., Renis M., Ientile R., Currò M., Caccamo D. (2016). Assessment of glutathione peroxidase-1 polymorphisms, oxidative stress and DNA damage in sensitivity-related illnesses. Life Sci..

[113] Dantoft T.M., Skovbjerg S., Andersson L., Claeson A.S., Engkilde K., Lind N., Nordin S., Hellgren L.I. (2017). Gene expression profiling in persons with multiple chemical sensitivity before and after a controlled n-butanol exposure session. BMJ Open..

[114] Cannata A., De Luca C., Korkina L.G., Ferlazzo N., Ientile R., Currò M., Andolina G., Caccamo D. (2020). The SNP rs2298383 reduces ADORA2A gene transcription and positively associates with cytokine production by peripheral blood mononuclear cells in patients with Multiple Chemical Sensitivity. Int. J. Mol. Sci..

[115] Ross G.H. (1992). Treatment options in multiple chemical sensitivity. Toxicol. Ind. Health.

[116] Driesen L., Patton R., John M. (2020). The impact of multiple chemical sensitivity on people’s social and occupational functioning; a systematic review of qualitative research studies. J. Psychosom. Res..

[117] Damiani G., Gironi L., Pacifico A., Cristaudo A., Malagoli P., Allocco F., Bragazzi N.L., Linder D.M., Santus P., Buja A. (2021). Masks use and facial dermatitis during COVID-19 outbreak: Is there a difference between CE and non-CE approved masks?. G Ital. Dermatol. Venereol..

[118] Damiani G., Gironi L.C., Grada A., Kridin K., Finelli R., Buja A., Bragazzi N.L., Pigatto P.D.M., Savoia P. (2021). COVID-19 related masks increase severity of both acne (maskne) and rosacea (mask rosacea): Multi-center, real-life, telemedical, and observational prospective study. Dermatol. Ther..

[119] Damiani G., Gironi L.C., Kridin K., Pacifico A., Buja A., Bragazzi N.L., Spalkowska M., Pigatto P.D.M., Santus P., Young Dermatologists Italian Network (2021). Mask-induced Koebner phenomenon and its clinical phenotypes: A multicenter, real-life study focusing on 873 dermatological consultations during COVID-19 pandemics. Dermatol. Ther..

